# Chemotherapy Options beyond the First Line in HER-Negative Metastatic Breast Cancer

**DOI:** 10.1155/2020/9645294

**Published:** 2020-11-28

**Authors:** Vito Lorusso, Agnese Latorre, Francesco Giotta

**Affiliations:** Medical Oncology Unit, IRCCS Istituto Tumori “Giovanni Paolo II”, Bari, Italy

## Abstract

Despite the recent advances in the biological understanding of breast cancer (BC), chemotherapy still represents a key component in the armamentarium for this disease. Different agents are available as mono-chemotherapy options in patients with locally advanced or metastatic BC (MBC) who progress after a first- and second-line treatment with anthracyclines and taxanes. However, no clear indication exists on what the best option is in some populations, such as heavily pretreated, elderly patients, triple-negative BC (TNBC), and those who do not respond to the first-line therapy. In this article, we summarize available literature evidence on different chemotherapy agents used beyond the first-line, in locally advanced or MBC patients, including rechallenge with anthracyclines and taxanes, antimetabolite and antimicrotubule agents, such as vinorelbine, capecitabine, eribulin, ixabepilone, and the newest developed agents, such as vinflunine, irinotecan, and etirinotecan.

## 1. Introduction

Breast cancer (BC) is the most commonly diagnosed form of cancer and the second leading cause of cancer-related death among women worldwide [1]. The estimates of the World Health Organization (WHO) reported over 2 million people diagnosed with BC worldwide in 2018 and over 600,000 annual deaths [2]. Most of the patients are initially diagnosed with stages I–III BC and may subsequently develop distant recurrence, whereas approximately 5-6% of women are affected by metastatic BC (MBC, stage IV) at the time of the first diagnosis [3]. Median overall survival (OS) in patients with MBC is approximately 37 months, with better results reported by women with hormone receptor positive (HR+) and human epidermal receptor 2 positive (HER2+) tumors (42.12 and 44.91 months, respectively) as compared with those with triple-negative BC (TNBC: 14.52 months) [4]. In a recent published paper, it was reported that young age appeared associated with a more aggressive clinical presentation of MBC, even if it did not impact the overall survival [5].

In the last decades, important advances have been made in the treatment of MBC, mainly thanks to a more comprehensive understanding of the molecular biology of the disease, which has prompted the development of more effective and individualized approaches [6]. Nevertheless, chemotherapy still represents a fundamental option for patients with MBC, regardless of the molecular subtype.

First-line treatment is usually represented by endocrine therapy (e.g., aromatase inhibitors and fulvestrant ±CDK 4/6 inhibitors) in case of HR-positive disease and by targeted therapies (e.g., trastuzumab/pertuzumab + chemotherapy) in patients with HER2-positive BC [7]. Chemotherapy may be introduced in cases of disease progression in HR-positive patients or it may be combined with target agents to treat HER2-positive disease. Moreover, despite the identification of promising targeted agents with encouraging results in therapy of TNBC, conventional chemotherapy still remains a valid systemic option in these patients [8].

Both anthracyclines and taxanes are used in patients with MBC, even if the latter are more often used in first line [9, 10]; however, they are not curative, and patients eventually develop drug resistance, one of the main obstacles towards a successful treatment [11]. Patients who experience disease progression are addressed to subsequent treatment lines, with approximately half of the women with MBC receiving overall >3 lines of chemotherapy [12]. Given the increasing proportion of pretreated or drug-refractory patients, it is a challenge to define which chemotherapy regimen is most active beyond the first line and especially in heavily pretreated patients [11]. A sequential strategy of multiple single agents seems to be preferable over the combinational approach, as the latter, despite achieving higher response rates, has little impact on OS and is associated with higher risk of toxicity [10, 13].

Carboplatin has been shown to increase the pathological complete response rate and to decrease the recurrence risk in neoadjuvant setting in triple-negative BC [14]. A recent meta-analysis of randomized trials assessing the efficacy and safety of platinum salts in woman with locally advanced or metastatic BC reported that this treatment significantly prolonged OS and PFS of patients with no unexpected toxicity [15]. In addition, a subpopulation of advanced triple-negative breast cancer patients with impairment of homologous recombination repair due to mutation in *BRCA1/2* would benefit from carboplatin treatment, as recently suggested by the TNT trial [16]. Atezolizumab, a PD-L1, inhibitor was recently approved by FDA for locally advanced or metastatic triple-negative breast cancer, positive for the expression of PD-L1 in combination with nab-paclitaxel. The approval was based on observed increased progression-free survival among patients with metastatic triple-negative breast cancer treated with atezolizumab plus nab-paclitaxel when compared to placebo plus nab-paclitaxel in the intent-to-treat (ITT) population and the PD-L1+ subgroup [17].

Another interesting aspect still under discussion is how elderly patients with MBC respond to chemotherapy. BC is largely a disease of older women, with a median age at diagnosis of 62 years [18] and increasing risk of developing BC in older patients (0.1% vs 3.9% for 20- vs 70-year-old women) [18]. The therapeutic advances in locoregional and systemic therapy have also improved patients' life expectancy, further increasing the proportion of elderly patients in need of treatment. Despite the increasing proportion of elderly MBC patients, no definitive evidence is available on which chemotherapy agent is most effective and tolerable in this frail population. Notably, treatment of older patients with multiple comorbidities, advanced disease, and prior exposure to multiple therapies tends to increase the risk of adverse events and may have significant negative effects on patients' functioning [19–21]. Defining the best chemotherapy approach in this population is particularly critical also considering that the key aims for MBC treatments are both increasing OS and improving quality of life (QoL).

In this paper, we reviewed literature evidence on the efficacy and safety of mono-chemotherapy regimens used beyond the first line in patients previously treated with anthracyclines and taxanes, focusing on specific populations and settings, such as elderly, TNBC subtypes, and heavily pretreated patients.

## 2. Current Chemotherapeutic Options in MBC after the First Line

### 2.1. Anthracyclines and Taxanes

Patients who progress after treatment with anthracyclines or taxanes may benefit from *rechallenge* with an already used or novel agent, although no clear comparative data are available on this topic [22] (Table 1).

#### 2.1.1. Doxorubicin

Pegylated doxorubicin (PLD) and nonpegylated doxorubicin (NPLD), as less cardio-toxic anthracyclines, may be useful in patients who received anthracycline-based adjuvant therapy with a long relapse-free interval, no significant cardiac impairment, and limited therapeutic options [23, 24]. In addition, these drug formulations allow higher drug dose than the cumulative lifetime dose of 550 mg/m^2^ of doxorubicin [32]. An overall response rate of 10–13% is reported with these agents in patients at their first relapse, with a PFS of 2–3.6 months, with possibility of prolonging therapy administration of anthracycline beyond the 6th cycle for a long time as maintenance treatment. However, no evidence is available to support PLD or NPLD rechallenge in later lines of therapy.

Even a recent phase I/II suggests that PLD combined with metronomic oral cyclophosphamide is an active and tolerable regimen in metastatic breast cancer [33], although its precise treatment role is still to be defined.

Epirubicin, a doxorubicin analog, has been shown to be equally active and less cardiotoxic than doxorubicin in metastatic breast cancer patients [34, 35].

#### 2.1.2. Docetaxel and Paclitaxel

Docetaxel and paclitaxel are the most commonly used taxanes in BC and are usually preferred in case of progression on a prior anthracycline regimen or in subjects who are at higher risk for cardiac toxicity and cannot benefit from this therapeutic option [36, 37].

In 2005, Jones et al. compared the safety and efficacy of the two taxanes in patients with advanced BC after progression on an anthracycline-containing regimen [25]. Docetaxel (100 mg/m^2^ every 3 weeks) reported superior results compared with paclitaxel (175 mg/m^2^ every 3 weeks) both in terms of median OS (15.4 vs 12.7 months; 95% CI: 1.15–1.73; *p*=0.03) and median time to progression (TTP; 5.7 v 3.6 months; 95% CI: 1.33–2.02; *p*=0.0001) and with numerically higher overall response rate (ORR; 32 vs 25%; *p*=0.10). Although docetaxel was associated with increased incidence of hematologic and nonhematologic adverse events (AEs), the toxicity profile was considered manageable and no significant differences were reported in the patients' QoL in the two treatment groups [25].

The Cancer and Leukaemia Group B (CALGB) protocol 9840 addressed if weekly paclitaxel (80 mg/m^2^) could be more effective and less toxic than paclitaxel 3-weekly 175 mg/m^2^ administration in MBC [38]. The data showed that the weekly paclitaxel schedule was superior to every-3-week administration with a higher response rate (42% vs 29%, 1.75; *p*=0.0004), time to progression (median, 9 vs 5 months; *p*=0.0001), and survival (median, 24 vs 12 months; *p*=0.0092); grade 3 neuropathy was more common with the weekly schedule (24% vs 12%; *p*=0.0003).

The 3-weekly schedule of paclitaxel and docetaxel may be unsuitable in elderly and/or frail patients due to the high risk of hematological toxicities [39]. Beuselinck et al. evaluated the feasibility of a reduced regimen consisting of weekly administration of paclitaxel 80 mg/m^2^ or docetaxel 36 mg/m^2^ in elderly patients unfit for the standard 3-weekly therapy. The study included patients ≥70 years classified as frail due to expected hematological problems, liver abnormalities, and grade 2 side effects associated with the 3-weekly administration. The weekly regimen with reduced dose proved to be more active, with partial response (PR) in 48% vs 38% of patients, stable disease (SD) in 24% vs 16%, median TTP of 21.1 vs 12.7 weeks, and median OS of 55.7 vs 32 weeks for paclitaxel and docetaxel, respectively. Both agents showed an acceptable toxicity profile; notably, paclitaxel was more frequently associated with anemia and neurotoxicity, while edema and fatigue were more frequent in the docetaxel group [26].

#### 2.1.3. Nab-Paclitaxel

Nab-paclitaxel is an albumin-based paclitaxel delivery system that showed superior response rates and improved tolerability profile over standard paclitaxel [40], and that obtained similar results compared with docetaxel [41], Blum et al. conducted a phase II trial on 181 women with MBC receiving either 100 mg/m^2^ or 125 mg/m^2^ nab-paclitaxel after progression on conventional taxanes therapy [27]. Both dosages showed similar antitumor activity, with an ORR of 14% vs 16% and SD ≥16 weeks in 12% and 21% of the patients, respectively. However, the lower dosage reported a better safety profile, with no hypersensitivity reactions, minimal myelosuppression, and few cases of treatment discontinuation due to peripheral neuropathy (PN) [27].

A subsequent retrospective analysis further confirmed the potential of nab-paclitaxel in the treatment of heavily pretreated taxane-refractory subjects, reporting encouraging ORR and PFS data and no major safety concerns [28–30, 42].

The improved efficacy and tolerability profile of nab-paclitaxel compared to conventional taxanes has also prompted the evaluation of this agent in elderly patients, with overall positive results for the weekly scheduled (150 mg/m^2^ for 3 weeks every 4 weeks) in subjects aged more than 65 years old [31, 43]. Interesting results came from a post hoc analysis of a phase II and a phase III trial, in which nab-paclitaxel (weekly and 3-weekly regimen) was compared to paclitaxel and docetaxel monotherapy in elderly patients with MBC (mean age ≥65 years old). The nab-paclitaxel weekly regimen was more active than the 3-weekly regimen, and it was associated with less serious AEs. However, the analysis of data from patients treated in the second-line setting and above, showed that paclitaxel had better ORR (14% vs 6%) and PFS (3.5 vs 2.1 months) compared with nab-paclitaxel [31].

Interestingly, nab-paclitaxel has shown promising results in the treatment of subjects with TNBC, with an ORR ranging from 34 to 85% [44–46]. However, in most of these studies, nab-paclitaxel was tested in combinational regimens with multiple agents including bevacizumab, gemcitabine, and carboplatin, and the investigations did not focus specifically on pretreated patients [44–46].

### 2.2. Non-Anthracycline and Taxane Chemotherapy Agents

The results of the main trials on vinorelbine, capecitabine, and eribulin are summarized in Table 2.

#### 2.2.1. Vinorelbine

Vinorelbine is a semisynthetic vinca alkaloid commonly used as second-line treatment or beyond in patients with MBC [70]. A recent real-world study evaluating the effect of vinorelbine in routine clinical practice showed that this agent was effective and well-tolerated as single agent in 55 patients with MBC who were previously treated with anthracyclines or taxanes, with median PFS of 3.7 months, median OS of 10 months, ORR of 29.1%, and clinical benefit in 49.1% of the patients.

However, vinorelbine as a single agent has not proven to be superior to other chemotherapeutic options in patients previously treated with anthracyclines and taxanes [48, 49]. A retrospective analysis comparing the effect of vinorelbine, capecitabine, or the combination of both agents in patients with MBC previously treated with anthracyclines and taxanes reported superior results for capecitabine both in terms of OS (188 days vs 102 days for vinorelbine) and 1-year survival rate (28.4% vs 15.6%) [48]. Similarly, another comparative study showed that docetaxel was associated with higher, but not statistically superior, PFS, OS, and ORR compared with vinorelbine in MBC patients progressing after an anthracycline treatment. Notably, vinorelbine had a remarkably superior safety profile with fewer significant grade 3–4 AEs, whereas docetaxel was associated with important hematological toxicity [49].

Different studies have also investigated the efficacy and safety of vinorelbine in combination with other agents, including gemcitabine, doxorubicin, epidoxorubicin, or capecitabine, reporting mixed results [71–75]. These combinations were rarely compared to vinorelbine alone in randomized studies and thus uncertainty still remains on real superiority of two drug combinations over vinorelbine as a single agent. However, an available option for patients pretreated with anthracyclines or taxanes seems to be the combination of vinorelbine plus gemcitabine, which reported superior PFS (6.0 months vs 4.0 months) and ORR (36% vs 26%) compared with vinorelbine alone [71].

Finally, multiple phase II studies have investigated the use of vinorelbine in a metronomic schedule, reporting a long-lasting disease control and a good toxicity profile [76–80].

#### 2.2.2. Capecitabine

Several phase II and some randomized phase III trials have proven the efficacy and safety of capecitabine as a single agent in pretreated MBC patients [27, 51, 52, 81–84]. In particular, two multicenter phase II studies have shown that capecitabine is an effective and well-tolerated option in patients with taxane-pretreated MBC, with ORR up to 26%, PFS up to 3.5 months, and OS up to 12.2 months [51, 52]. A systematic review published in 2011 analyzed the data of 1494 patients treated with capecitabine, of which 80% were pretreated with anthracyclines and taxanes, further confirming the efficacy of this drug in the second line with ORR 18%, median PFS 4.2 months, and OS 13.5 months [85]. Of note, similar efficacy results have been reported in multiple randomized controlled trials comparing capecitabine monotherapy with different combinations including docetaxel, vinflunine, ixapebilone, utidelone, and irinotecan [86–91].

No clear evidence is available on the effects of capecitabine beyond the first line in MBC patients with different tumor biology, although an analysis conducted by Glück et al. suggests that capecitabine might be more effective in the HR-positive subtype group [92].

Two studies have explored the use of capecitabine in older MBC patients. In 2004, Bajetta et al. evaluated the efficacy and safety of two doses of capecitabine (1250 mg/m^2^ or 1000 mg/m^2^ twice daily on days 1–14 every 21 days) in 73 older women (mean age: 73 years old, range 65–89) with MBC [53]. Although the study did not focus specifically on second-line treatment, 60% of the subjects enrolled had received prior neo/adjuvant chemotherapy. The study reported similar response rates in the two groups (36.7% and 34.9% for the standard and low-dose regimen, respectively) and a good tolerability profile, with low overall incidence of grade 3/4 AEs [53].

In 2011, Blum et al. conducted a pooled analysis of five phase II and III trials on capecitabine to determine if an association existed between patients' age and treatment efficacy [93]. Of 570 patients, 193 (34%) were 18–49 years old, 246 (43%) were 50–64 years old, and 131 (23%) were ≤65 years old. Overall survival in all groups was similar by the log-rank test for the individual trials (*p*=0.71 − 0.95) and Cox regression of the pooled trials. Univariate analysis demonstrated no difference in clinical benefit or objective response between groups. Serious AEs occurred in 71 (36.8%), 85 (34.6%), and 59 (45.0%) patients in the 18–49, 50–64, and >65 years groups, respectively. The study concluded that no statistically significant association was observed, respectively, for age and OS, clinical benefit, or objective response in patients with MBC treated with capecitabine. Moreover, the frequency of AEs and serious AEs was not related to age at treatment, although women >65 years were more likely to discontinue treatment due to safety reason as compared with younger women [93].

Of note, analysis of genetic variants of enzymes involved in capecitabine metabolism (i.e., dihydropyrimidine dehydrogenase-DPYD) should be performed as some specific *DPYD* polymorphisms have been associated with both drug related toxicity and efficacy [94].

#### 2.2.3. Eribulin

Eribulin, a synthetic analog of the natural molecule halicondrin, is a nontaxane microtubule dynamics inhibitor [95]. The approval of eribulin was based on the results of the registrative phase III trial EMBRACE (Eisai Metastatic BReast Cancer study Assessing physician's Choice vs E7389), which showed the superiority of eribulin over “treatment of physicians' choice” (TPC) in patients with locally recurrent or MBC previously treated with two to five chemotherapy regimens, including anthracyclines and taxanes [56]. The trial reported a statistically significant increase in OS of 2.7 months with eribulin vs TPC, positive results in terms of TTP and ORR, a good tolerability profile with the most common grade 3-4 AEs being neutropenia, and limited cases of febrile neutropenia and peripheral neuropathy. Among the nonheamatologial toxicities, alopecia of any grade has been reported in 45% of the treated patients [56].

The efficacy and safety profiles of eribulin are overall comparable to that of capecitabine, as shown by Kaufman et al. in 2015 in a phase III randomized trial in women with MBC previously treated with anthracyclines and taxanes [60]. The study showed that the two options were comparable in terms of ORR (11% vs 11.5% for eribulin and capecitabine, respectively), OS (15.9 vs 14.5 months), and PFS (4.1 vs 4.2 months), and that both treatments had a manageable safety profile, with similar results also in terms of global health status and QoL [60].

Data from the EMBRACE trial [56] and the comparative study of Kaufman et al. [60] were subsequently analyzed as specifically requested by EMA to allow a more specific evaluation of eribulin activity in different subgroups. The results of the pooled analysis confirmed that eribulin prolonged OS by 2.4 months (hazard ratio (HR): 0.85; 95% CI: 0.77–0.95; *p*=0.003) over the control arm, with a favorable profile in all the subgroups and in particular in patients with HER2-negative disease (HR: 0.82; 95% CI: 0.72–0.93) [96].

Two *post hoc* analyses further explored the comparative results of eribulin and capecitabine in different subgroups of patients [61, 97]. The analysis conducted by Twelves in 2016 showed that, despite similar results in PFS, eribulin prolonged the OS over capecitabine in various subgroups, including HER2-negative patients (15.9 vs 13.5 months, respectively), HR-negative patients (14.4 vs 10.5 months), and TNBC (14.4 vs 9.4 months) [97]. Similar results were obtained in 2017 by Pivot et al. [61], who repeated the analysis focusing only on 392 patients with HER2-negative disease, including HER2-negative/HR-positive and TNBC treated in the second-line setting. The results showed an improved OS in patients receiving eribulin (16.1 months) compared with capecitabine (13.5 months), although no major changes were observed in terms of PFS (4.2 vs 4 months) and ORR (9.7% vs 9.7%; *p* = 0.86) [61].

The efficacy of eribulin in pretreated patients was further confirmed by the results of an expanded access program conducted in Belgium in a real-life setting [62]. Patients included in the study (*n* = 154) had received a mean number of four prior chemotherapy regimens, including anthracyclines, taxanes, and capecitabine and reported a mean OS of 11.3 months, mean PFS of 3.2 months, and ORR of 24% in favor of eribulin. The most common AEs were fatigue/asthenia (74%), alopecia (55%), peripheral neuropathy (46%), and neutropenia (43%). According to the subgroup analysis, the best response was achieved in HR-positive HER2-negative subjects, reporting 29% of ORR; TNBC obtained a similar response (21%), whereas the response of HER2-positive subjects was only 14% [62].

In 2014, Muss et al. [59] conducted an exploratory analysis on pooled data from two phase II and one phase III study to explore the correlation between age and response to eribulin treatment [59]. The analysis, including 827 patients divided in age groups (<50 years, *n* = 253; 50–59 years, *n* = 289; 60–69 years, *n* = 206; ≥70 years, *n* = 79), showed no significant effect of age on OS (11.8 months, 12.3 months, 11.7 months, and 12.5 months, respectively; *p* = 0.82), PFS (3.5 months, 2.9 months, 3.8 months, and 4.0 months, respectively; *p* = 0.42), ORR (12.7%, 12.5%, 6.3%, and 10.1%, respectively), or clinical benefit rate (CBR) (20.2%, 20.8%, 20.4%, and 21.5%, respectively). Moreover, eribulin showed a similar tolerability profile among younger and older patients, with no significant changes in the incidence of AEs in different age groups.

A recent observational study promoted by the Italian Group for Geriatric Oncology also focused on the elderly population by investigating the efficacy and safety of eribulin as third or subsequent line of chemotherapy in 50 heavily pretreated elderly patients with locally recurrent/MBC [98]. The study reported a median PFS of 4.49 months, median OS of 7.31 months; 51% of subjects obtained clinical benefit and only mild toxicities. QoL, measured through the comprehensive geriatric assessment (CGA) and health-related QoL, was not affected or worsened by eribulin. No significant changes were measured in terms of EQ-5D and EQ-5D-3L VAS; however, a progressive decrease was reported in instrumental activities of daily living score and in the percentage of patients who experienced no problems in daily activities, whereas the percentage of subjects referring minor problems in daily living and the Geriatric Depression Scale score tended to increase [98]. Some studies focused specifically on the efficacy and safety of eribulin in MBC patients with well-defined taxanes resistance [63, 65]. A phase II multicenter study conducted by Inoue et al. demonstrated the eribulin activity in heavily pretreated MBC patients resistant to taxanes [63]. The study, which included 51 Japanese female patients with taxane-resistant MBC and a median of four prior chemotherapy treatments, reported a median PFS of 3.6 months (95% CI: 2.6–4.6), median OS of 11.7 months (95% CI: 9.2–14.2), clinical benefit rate (CBR) of 39.2%, and PD of 49.0%. In this population, eribulin displayed a clinically manageable safety profile, with leukopenia (23.5%), neutropenia (35.3%), anemia (5.9%), and febrile neutropenia (7.8%) as the most common grade 3–4 AEs [63].

More recently, Lorusso et al. conducted a subanalysis on data extracted from the ESEMPIO database focusing on 91 subjects with well-defined taxane refractoriness [65]. The analysis showed that eribulin is effective and well tolerated in this particular population. In fact, the authors reported a clinical benefit in 45.2% of the subjects, with median PFS of 3.1 months and median OS of 11.6 months. The most commonly reported AEs were asthenia/fatigue (58%) and neutropenia (30%) [65].

Interestingly, eribulin seems to be an effective treatment also for male patients with BC, a population with high need of therapeutic options due to lack of evidence on their management and treatment [99]. This study shows the results of a retrospective multicenter study on a small group of 23 male patients treated with eribulin reported positive results, with all the patients reaching at least stable disease, two cases of complete response, and an overall well-tolerated safety profile [99].

#### 2.2.4. Ixabepilone

Ixabepilone, a semisynthetic analog of epothilone B that acts as microtubule inhibitor, is approved by the US Food and Drug Administration (FDA) as a single agent in patients with locally advanced or MBC who progressed after anthracyclines, taxanes, and capecitabine [100]. Notably, EMA has not granted the marketing authorization to ixabepilone due to concerns about its therapeutic index and especially the risk of neuropathy [101].

Multiple phase II clinical trials have reported the beneficial effects of ixabepilone monotherapy in patients with MBC or locally advanced disease [57, 67, 68].

In 2007, Thomas et al. evaluated the safety and efficacy of ixabepilone 40 mg/m^2^ 3-weekly monotherapy in 49 taxane-refractory patients [68]. The study reported on ORR of 12% (95% CI: 4.7–26.5%), median TTP of 2.2 months (95% CI: 1.4–3.2 months), and median OS of 7.9 months. From the safety point of view, patients reported primarily grade 1/2 and manageable AEs and mostly mild-to-moderate treatment-related neuropathy [68]. Another study conducted by Aogi et al. reported positive efficacy and safety results for ixabepilone in taxane-resistant MBC previously treated with anthracyclines [57]. Treatment with ixabepilone monotherapy resulted in mean ORR of 11.5% (95% CI: 4.4–23.4), TTP of 2.8 months and manageable toxicities with the most frequent grade 3–4 AEs being neutropenia (82.7%), leukopenia (75%), myalgia (19.2%), and peripheral neuropathy (19.2%) [57].

Ixabepilone monotherapy also showed clinical activity in a group of 126 patients resistant to anthracycline, taxane, and capecitabine [67]. Patients were heavily pretreated, with 88% having received at least two lines of prior chemotherapy in the metastatic setting. ORR was 11.5%, with 50% of the patients achieving SD and 14.3% with SD ≥6 months; median PFS was 3.1 months (95% CI: 2.7–4.2 months), and median OS was 8.6 months (95% CI: 6.9–11.1 month). Grade 3/4 treatment-related AEs included peripheral sensory neuropathy (14%), fatigue/asthenia (13%), myalgia (8%), and stomatitis/mucositis (6%).

Although the approved dose of ixabepilone is 40 mg/m^2^ 3-weekly, this therapeutic regimen may require dose reductions and delays in response due to the occurrence of AEs such as myelosuppression, peripheral sensory neuropathy, fatigue/asthenia, arthralgia/myalgia, and gastrointestinal disturbance [67, 68]. However, a study conducted by Smith et al. in 2013 [69] does not suggest that a reduced regimen of ixabepilone (16 mg/m^2^ weekly) is as effective as the standard 3-weekly schedule of 40 mg/m^2^. Indeed, the comparative study showed significantly prolonged median PFS with the 3-weekly schedule (5.3 vs 2.9 months; log-rank *p*=0.05) compared with the weekly regimen. Despite the superior efficacy, the higher dose was associated with increased rates of grade 3–4 AEs, particularly neutropenia (38.2 vs. 6.1%), and a higher frequency of toxicity-related discontinuations [69].

As suggested by a phase II comparative study, ixabepilone seems to be less effective than eribulin in patients with MBC or locally recurrent disease previously treated with taxanes and another chemotherapy regimen [58]. The study, conducted in 101 patients, showed superior results for eribulin vs ixabepilone both in terms of ORR (15.4% vs 5.8%) and PFS (104 vs 95 days) and reported a better safety profile especially in terms of occurrence of peripheral neuropathy, a common AE reported with tubulin-target chemotherapy. In fact, eribulin was associated with a reduced incidence of neuropathy (33.0 vs 48.0% for any grade and 9.8% vs 22% for grade 3–4) and peripheral neuropathy (31.4% vs 44% for any grade and 9.8% vs 20% for grade 3–4) compared with ixabepilone. However, the different incidences of neuropathy with the two regimens did not reach statistical significance when results were adjusted for preexisting neuropathy and number of prior chemotherapies [58].

Lastly, a systematic review conducted in 2016 summarized the results of eight randomized controlled trials that investigated the safety and efficacy of ixabepilone alone or in combination with other treatments in MBC [102]. Overall, the results suggest that a 3-weekly schedule of ixabepilone is more effective than weekly dosing in improving ORR and that the combination of ixabepilone and capecitabine possesses superior clinical efficacy to capecitabine alone. In terms of comparison with other agents, paclitaxel seems to be more effective than ixabepilone in terms of OS and PFS, whereas the efficacy and safety of ixabepilone and eribulin are comparable [102].

Despite the existence of multiple evidence on ixabepilone in MBC, its role as single-agent treatment in this population is still unclear, especially considering the risk-benefit evaluation.

### 2.3. Newest Agents

The results of the main trials of newest agents for locally advanced or MBC are summarized in Table 3.

#### 2.3.1. Vinflunine

Vinflunine is a third-generation fluorinated vinca alkaloid currently under investigation for the treatment of MBC beyond anthracycline-/taxane-based chemotherapy. Two phase II trials have reported encouraging results in this population, with ORRs ranging from 12.5% to 30%, median PFS from 2.6 to 3.7 months, and OS from 11.4 to 14 months [103, 104].

Based on the results of a phase II study conducted on 38 patients refractory/resistant to vinorelbine, vinflunine seems to be a safe and effective option in MBC patients previously treated with vinorelbine [105]. Vinflunine showed a positive safety profile in this population, with the most common AEs being neutropenia (75.6%), fatigue (44.7%), constipation (28.9%), and abdominal pain (26.3%) and no case of dose reduction due to toxicities. In terms of efficacy, vinflunine showed an antitumor activity with ORR of 8.3% (95% CI: 1.75–22.4), PFS of 4.0 months (95% CI: 2.5–6.1), and OS of 13.6 months (95% CI: 8.7–18.9) [105].

An open-label phase III trial published in 2018 also showed that vinflunine is as effective as other alkylating agents in the treatment of heavily pretreated MBC patients (at least two prior chemotherapy regimens including anthracycline, taxane, antimetabolite, and vinca alkaloid), thus suggesting the need of further evaluations on this molecule [106]. No difference was measured between vinflunine and the other treatment arms in terms of OS (median 9.1 vs 9.3 months), PFS (median 2.5 vs 1.9 months), or ORR (6% vs 4%). Moreover, the safety profile of vinflunine was acceptable, with neutropenia (19%) and asthenia (10%) as the most common grade 3–4 AEs [106].

Vinflunine associated with capecitabine in a phase III trial in patients with anthracycline- and taxane-pretreated locally recurrent/metastatic breast cancer demonstrated a modest improvement in PFS and an acceptable safety profile compared with capecitabine alone [86]. However, availability issues exist for this drug, as it is available only in France.

#### 2.3.2. Irinotecan

Irinotecan is a topoisomerase I (Top1) inhibitory prodrug that exerts its cytotoxic effect by disrupting DNA replication after being enzymatically converted to the active metabolite SN-38 [110]. Irinotecan administered at the dose of 240 mg/m^2^ every 3 weeks has shown clinical activity in MBC patients pretreated with anthracyclines and taxanes, although the occurrence of important AEs, including neutropenia, diarrhea, and nausea/vomiting, has raised some concerns over its safety profile [111, 112].

Perez et al. reported a superior activity and better tolerability for the weekly 100 mg/m^2^ dose compared to the 3-weekly regimen in terms of ORR (23% vs 14%) and median PFS (2.8 vs 1.9 months) [112]. Another study conducted by Hayashi et al. evaluated the effects of a biweekly administration of irinotecan 150 mg/m^2^, focusing on HER2-negative MBC patients previously treated with anthracyclines and taxanes in particular [107]. The biweekly administration proved to be feasible in this population with ORR of 5.6%, median PFS of 3.2 months, and median OS of 9.6 months. The safety profile was also acceptable with no treatment-related deaths and grade 3–4 neutropenia (22.2%), anorexia (11.2%), diarrhea (11.2%), and fatigue (5.6%) as the most common AEs.

#### 2.3.3. Etirinotecan

In order to increase the presence of SN-38 (the active metabolite) at the site of the tumor, a new molecule called etirinotecan has been developed starting from irinotecan. The new agent is a long-acting polymer-engineered molecule comprising irinotecan bound to a proprietary polyethylene glycol core by a biodegradable linker that slowly hydrolyzes in vivo to release the active metabolite SN-38.

Etirinotecan has shown promising results in the treatment of MBC patients previously treated with taxane and receiving ≤2 previous chemotherapy regimens. A phase II open-label study on etirinotecan pegol 145 mg/m^2^ administered every 2 weeks (q14d) or every 3 weeks (q21d) showed positive efficacy results, with ORR up to 29% (95% CI: 18.4–40.6), PFS up to 5.6 months (95% CI: 2.7–5.7 months), and OS up to 13.1% [108]. Etirinotecan also showed a significantly different safety profile compared to irinotecan, with the most serious toxicity being delayed diarrhea (q14d: 69% all grades, 20% grade 3–4; q21d: 77% all grades, 23% grade 3, no grade 4). The subset analysis further confirmed that etirinotecan, especially in the 3-weekly setting, was active in patients with TNBC and in subjects who had previously received anthracyclines, taxanes, and capecitabine [108].

Perez et al. compared the efficacy and safety of etirinotecan 3-weekly with treatment of physician's choice in a multicenter randomized open-label phase III trial [109]. This trial did not demonstrate an improvement in OS for etirinotecan pegol compared to TPC (12.4 vs 10.3 months) in patients with heavily pretreated MBC. However, etirinotecan seemed to be particularly beneficial for patients with brain metastases (*n* = 67), which reported an OS advantage of 10.0 months (vs 4.8 for TPC) and 12-month OS of 44.4% (vs 19.4% for TPC) and in those with liver metastases (*n* = 456) who reported increased OS of 10.9 months (vs 8.3 for TPC) [109]. As previously described for vinflunine, accessibility issues exist even for this drug that it is still unavailable.

### 2.4. Role of Chemotherapeutic Agents in Different Population Settings

#### 2.4.1. Refractory Patients

Literature data show that both capecitabine and eribulin are effective and well-tolerated in patients who progress after a taxane and anthracycline regimen.

A phase II study reported the tolerability and effectiveness of capecitabine in taxane-refractory or taxane failing MBC with ORR up to 26% [51].

Eribulin has also proven to be an excellent option in this population, reporting a tolerable safety profile and remarkable effectiveness in heavily pretreated taxane-refractory patients with a response rate of 18.6% and overall disease control in approximately half of the patients (46%) [65]. Other studies have reported the effects of eribulin in this population, with 37.7% of complete/partial responses and 48.9% of clinical benefit [54, 55]. Moreover, according to a multivariate analysis, primary taxane resistance is an independent predictive factor for clinical benefit and an independent prognostic factor for TTP in patients treated with eribulin [113].

The role of vinorelbine and ixabepilone single agent treatment of in-patients previously treated with anthracyclines and taxanes has yet to be defined in terms of efficacy and safety, as suggested in several studies [48, 49, 57, 67].

#### 2.4.2. Triple-Negative Patients

Although no clear indication exists on which subgroup of patients better responds to capecitabine, according to Glück et al., this agent seems to be most effective in patients with HR-positive disease [92].

Hints of activity of iritontecan in combination with capecitabine have been reported in TNBC [114, 115].

In a phase II study of the irinotecan + capecitabine combination in 36 MBC patients previously treated with anthracycline and taxane chemotherapy, 4 out of 8 patients with TNBC achieved a partial response.

The comparative analyses of pooled data from the EMBRACE trial and the phase III randomized study by Kaufman et al. have shown that eribulin is superior to capecitabine in the subgroups of patients with HER2-negative disease, which includes subjects with TNBC [56, 60, 96]. This observation has been further confirmed by a post hoc analysis conducted in 2016, which reported the superiority of eribulin over capecitabine in HER2-negative (OS of 15.9 vs 13.5, respectively), HR-negative (14.4 vs 10.5 months), and TBNC patients (14.4 vs 9.4 months) [97] and by an analysis specifically conducted on HER2-negative subjects, in which eribulin proved to prolong OS compared with capecitabine (16.1 vs 13.5 months) despite not reaching a statistically significant improvement in PFS and OS [61]. The efficacy of eribulin in the triple-negative sub-group was also observed in the real-life setting, as reported in the Belgium-expanded access program. The subgroup analysis showed that eribulin was most effective in HER2-negative ER-positive patients (ORR 29%) followed by TNBC patients (21%). Notably, patients in this study had already been treated with both anthracyclines and taxanes and capecitabine [62].

Perez et al. summarized the efficacy and safety of ixabepilone in TNBC patients [116]. The revised data from 7 trials suggested activity of the drug in this setting across multiple disease settings, from neoadjuvant to refractory MBC. Ixabepilone seemed to have better clinical outcomes in TNBC patients compared with those with receptor-positive breast cancer, with comparable toxicity.

#### 2.4.3. Elderly Patients

Elderly patients represent a frail population, and there is evidence that treatment of older patients with multiple comorbidities and prior exposure to multiple therapies increase the risk of adverse events [19–21]. In addition, older patients are generally poorly represented in clinical trials [117]. Nevertheless, some data have been published on treatment beyond first line in this MBC patient subset. The response to both eribulin and capecitabine appears to be independent from patients' age, with similar efficacy in different age groups and no major differences in the safety profile between younger and older subjects [59, 93]. However, as reported by Blum et al., patients aged > 65 years treated with capecitabine seem to suffer from a higher percentage of treatment discontinuation compared to younger subjects [93]. Different studies have reported the efficacy and safety of eribulin in previously treated older patients [59, 98]. The Italian Group for Geriatric Oncology has shown that eribulin not only was safe and effective in patients >65 years old but also preserved QoL in this particularly frail population [98]. QoL is recognized, together with increasing OS, as one of the main objectives of MBC treatment. Of note, eribulin has shown tolerable safety profile and OS up to 11.7 months in heavily pretreated patients, including subjects previously treated with anthracyclines, taxanes and also capecitabine [57, 58, 63]. Given the treatment advances and the improved life expectancy of patients, it is extremely common for elderly patients to have received multiple previous treatment lines, thus further justifying the use of eribulin in this particularly challenging population.

## 3. Conclusions

**Figure 1:**
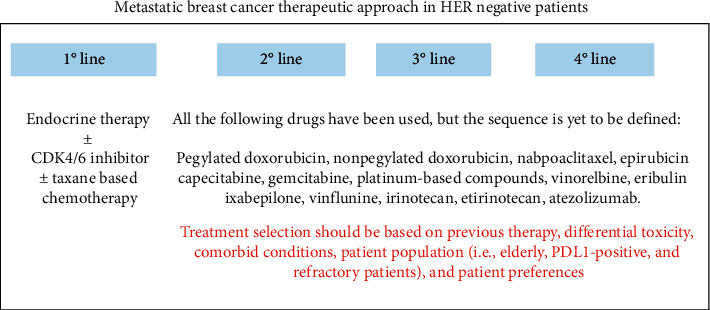
Schematic metastatic breast cancer therapeutic approach in HER-negative patients.

Anthracyclines and taxanes are commonly used as a first-line chemotherapy regimen in patients with MBC [9]. This therapy is however not curative and drug resistance will eventually develop. Patients progressing are offered subsequent treatment lines, with approximately half of the women with MBC receiving overall >3 lines of chemotherapy [12]. It has been suggested that sequential strategy of multiple single agents seems to be preferable over the combinational approach, as this latter approach has little impact on OS and is associated with higher risk of toxicity [13] (Figure 1). In addition, as nicely discussed and recommended by Partridge et al. [118], the choice of treatment in this patient population should rely not only on efficacy data but also on other factors such as treatment side effects, patient performance status, the presence of comorbidities, and patient personal preference.

Among the newest agents, vinorelbine has shown to be safe and effective in MBC; however, this agent is commonly used in combinational regimens contributing to increased toxicity, which can be very challenging in elderly subjects [48, 49, 75]. Ixabepilone, despite being a feasible option for MBC, has some safety concerns, especially in the 3-weekly regimen [68, 101]. In addition, based on literature evidence, eribulin seems to be a very promising options as second- or further-line chemotherapy in patients with locally advanced or MBC who progress after one chemotherapeutic regimen for advanced disease.

## Figures and Tables

**Table 1:** *Rechallenge* with anthracycline or taxanes.

Author (study phase)	Treatment	Patients (*n*)	Patients age (years), median (range)	Triple-negative status	Drug resistance	Prior treatments	Efficacy			Safety (grade 3/4 AE)	References
							ORR	PFS	OS		
Doxorubicin											
Al Batran 2006 (phase II)	PLD 50 mg/m^2^ q4w	79	58 (35–79)	Unknown	A	A, T ≥ 3 prior CT (30%)	12.7%	3.6 mo	12.3 mo	Neutropenia 17.1%; leukopenia 14.4%	[23]
Keller 2004 (phase III)	PLD vs comparator (vinorelbine or mitomicin C + vinblastine)	150 vs 151	56 (33–87) vs 56 (30–83)	Unknown	A (39% vs 35%)	≥2 prior CT (38% vs 43%)	10% vs 12%	2.9 vs 2.5 mo	11.0 vs 9.0 mo	PLD: PPE 19%; stomatitis 5%; comparator: neutropenia 8%	[24]

Paclitaxel											
Jones 2005 (phase III)	Paclitaxel 175 mg/m^2^ vs docetaxel 100 mg/m^2^ day 1 q3w	224 vs 225	54 (28–82) vs 56 (22–93)	Unknown	Unknown	A (98.2%)	25% vs 32%	3.6 vs 5.7 mo	12.7 vs 15.4 mo	Neutropenia 54.4% vs 93.3%; FN 1.8% vs 14.9%; anemia 7.3% vs 10.4%	[25]
Beuselink 2010 (phase II)	Paclitaxel vs docetaxel	33 vs 37	<70 (*n* = 21 vs 21) ≥70 (*n* = 12 vs 16)	Unknown	Unknown	A (100% vs 97%), T (9% vs 5%), ≥2 prior CT (33% vs 35%)	48% vs 38%	TTP 21.1 vs 12.7 weeks	55.7 vs 32.0 weeks	Discontinuation 36% vs 45%; neutropenia 45% vs 20%; infection 15% vs 11%; stomatitis 3% vs 16%	[26]

Nab-paclitaxel											
Blum 2007 (phase II)	100 mg/m^2^ vs 125 mg/m^2^	10675	<65 (*n* = 86 vs 56) ≥65 (*n* = 20 vs 19)	Unknown	T (89% vs 78%)	Mean no. of prior MBC 3 (0–7) vs 3 (1–14)	14% vs 16%	3 vs 3.5 mo	9.2 vs 9.1 mo	Neutropenia: 18% vs 34% PN: 8% vs 19%	[27]
Dent 2013 (retrospective)	260 mg/m^2^ day 1 q3w vs 125 mg/m^2^ days 1, 8, 15 q4w	21 vs 21	57 (34–74) vs 34–74	Unknown	Unknown	Median 3 (range 1–6)	4.7% vs 14.2%	Unknown	13.6 vs 10.8 mo	SN 11.6%	[28]
Palumbo 2015 (phase II)	260 mg/m^2^ day 1 q3w	52	53 (33–71)	Unknown	Unknown	1 prior line: T (46.2%) or other agents (53.8%)	48.1%	8.9 months	Unknown	Neutropenia 21.2%; leukopenia 25%; PN 5.8%	[29]
Fabi 2015 (phase II)	260 mg/m^2^ d1q3w vs 125 mg/m^2^ d1, 8, 15 q4w	42	48 (21–80)	19%	Unknown	T (100%), A (85.8%) ≥ 2 lines (83.3%)	23.8%	4.6 months	Unknown	PN 12%, neutropenia 70% fatigue 13%	[30]
Aapro 2011 (post-hoc analysis)	Nab-paclitaxel 150–100 mg/m^2^ weekly vs 3-weekly vs sb-docetaxel	52 (phase II study)	≥65 years old	Unknown	Unknown	Prior chemotherapy from 30 to 84%	60–64% vs 22% vs 32%	18.9–9.2 vs 13.8 vs 8.5 mo	20.7–21.7 vs 19.9 vs 21.2 mo		[31]
	nab-paclitaxel 3-weekly vs sb-paclitaxel	62 (phase III study)					27 vs 19%	5.6 vs 3.5 mo	17.6 vs 12.8 mo		

PDL: pegylated doxorubicin; CT: chemotherapy; PPE: palmar-plantar erythrodysesthesia; FN: febrile neutropenia: mo: months; no: number.

**Table 2:** Antimetabolite and antimicrotubule agents.

Author (study phase)	Treatment	Patients (*n*)	Patients mean age (years) (range)	Triple-negative status	A, T, C resistance	Prior treatments	Efficacy			Safety (grade 3/4 ADR)	References
							ORR (%)	PFS	OS		
Vinorelbine											
Seo 2011 (phase II)	25 mg/m^2^ d1; 8, 15, 22 q4w	26	47 (37–71)	Unknown	Unknown	A, T	20.8	3.7 mo	10.4 mo	Neutropenia 69.2%, anemia 15.3%	[47]
Verma 2007	Vinorelbine vs capecitabine	45 vs 68	52 (41.1–62.9) vs 53 (43.5–62.5)	Unknown	Unknown	A, T	Unknown	Unknown	102 days vs 188 days	Unknown	[48]
Palmieri 2012	Vinorelbine 25 mg/m^2^ day 1 q2W vs docetaxel	18 vs 17	45 (33–67)	Unknown	Unknown	A: median (range): 2 (1–3)	6 vs 12.5	7.6 vs 10.4 weeks	21.2 vs 34 weeks	Neutropenia (*n* = 1 vs 11), fatigue (*n* = 2 vs 5), infection (*n* = 0 vs 7)	[49]
Blancas 2019 (real-world)	25 mg/m^2^ (d1), 8, 15, 22 q4w	55	67 (38–82)	25.5%	Unknown	A, T	29.1	3.7 mo	10 mo	Neutropenia 9.1%, FN 3.6%, constipation 3.6%	[50]

Capecitabine											
Blum 2001 (phase II)	1255 mg/m^2^ tid d1-14 q21d	74	52 (41–63)	Unknown	Unknown	T (100%) A (96%) ≥2 prior regimens (97.9%)	26	3.2 mo	12.2 mo	Hand-foot syndrome (22%), diarrhea (16%), stomatitis (12%)	[51]
Reichardt 2003 (Phase II)	1250 mg/m^2^ tid d1-14 q21d (with possible dose reductions)	136	56 (32–77)	Unknown	Unknown	T (99%), A (93%); mean: 2 (range 1–6)	15	3.5 mo	10.1 mo	Hand-foot syndrome (13%), diarrhea (8%), vomiting (4%), nausea (3%)	[52]
Bajetta 2005 (phase II)	1250 mg/m^2^ vs 1000 mg/m^2^ tid days 1–14 q21d	73	73 (65–89)	Unknown	Unknown		36.7 vs 34.9	4 vs 4 mo	Unknown	Diarrhea 13% vs 2%, dyspnea 10% vs 5%, fatigue 7% vs 12%, nausea 7% vs 5%	[53]

Eribulin											
Vahdat 2009 (phase II)	1.4 mg/m^2^; days 1, 8, 15 q28d vs days 1, 8 q21d	70 vs 33	55 (34–84) vs 52 (32–81)	27% vs 33%	Unknown	A, T; mean: 4 (1–11)	11.5	2.6 mo	9.2 mo	Neutropenia 64%, leukopenia 18%, fatigue 5%, PN 5%; FN 4%	[54]
Cortes 2010 (phase II)	1.4 mg/m^2^; days 1, 8 q21d	269	56 (26–80)	20%	A-T-C (9%)	A, T, C; mean: 4 (2–5)	9.3	2.6 mo	10.4 mo	Neutropenia: 54%, FN: 5.5%, PN: 6.9%	[55]
Cortes 2011 (phase II)	1.4 mg/m^2^; days 1, 8 q21d vs TPC	508 vs 254	55 (28–85) vs 56 (27–81)	18% vs 20%	A (56% vs 61%), T (81% vs 80%), C (67% vs 69%)	A, T, C (73%); mean 4 (1–7)	12 vs 5 (*p*=0.0002)	3.7 vs 2.2 mo (*p*=0.137)	13.1 vs 10.6 mo (*p*=0.041)	Neutropenia: 45% vs 21%, FN: 5% vs 2%, PN: 8% vs 2%	[56]
Aogi 2013 (phase II)	1.4 mg/m^2^; d1, 8 q21d	80	54.0 (31–72)	27.5%	Unknown	A, T, C (57.5%)	21.3	3.7 mo	11.1 mo	Neutropenia 95.1%, leukopenia 74.1%, FN 13.6%, PN 3.7%	[57]
Vahdat 2013 (phase II)	Eribulin 1.4 mg/m^2^; days 1, 8 q21d vs ixabepilone 40 mg/m^2^ q21d	51 vs 50	52.2 (42–62) vs 56.9 (46.2–67.5)	7.8% vs 22%	Unknown	>3 prior therapies 27.5% vs 38%	15.4 vs 5.8	104 vs 95 days	Unknown	Neuropathy 9.8 vs 22%; PN 9.8% vs 20%	[58]
Muss 2014 (pooled analysis)	1.4 mg/m^2^; days 1, 8 q21d	827	<50 (*n* = 253) 50–59 (*n* = 289) 60–69 (*n* = 206) ≥70 (*n* = 79)	22.9% 17.6% 20.9% 12.7%	Overall data A (27%), T (50.5%), C (65%)	A, T ≥ 2 prior regimens (99.3%)	12.7 12.5 6.3 10.1	3.5 mo 2.9 mo 3.8 mo 4.0 mo	11.8 mo 12.3 mo 11.7 mo 12.5 mo	Neutropenia: 43.9% vs 50.2% vs 52.9% vs 49.4%	[59]
Kaufman 2015 (phase III)	Eribulin 1.4 mg/m^2^; d1, 8 q21d vs capecitabine 1.25 g/m^2^ twice (d1) 14 q21d	554 vs 548	54 (24–80) vs 53 (26–80)	27.1% vs 24.5%	A (24.2% vs 25.4%), T (45.1% vs 47.4%)	A, T ≥ 2 prior MBC regimen (73.3% vs 72.1%)	11 vs 12	4.1 vs 4.2 mo (*p*=0.3)	15.9 vs 14.5 mo (*p*=0.056)	Neutropenia: 46% vs 4%; FN: 2% vs <1%; PN: 4% vs <1%; HFS: 0% vs 14%	[60]
Pivot 2018 (sub-group analysis)	Eribulin 1.4 mg/m^2^; days 1, 8 q21d vs capecitabine 1.25 g/m^2^ twice days 1–14 q21d	186 vs 206	≤40 (8.6% vs 17.5%) 40–65 (72.6% vs 72.8%) ≥65 (18.8% vs 9.7%)	39.2% vs 35%	Unknown	A, T	9.7% vs 8.7%	4.2 vs 4 mo	16.1 vs 13.5 mo	Neutropenia: 43.5% vs 5.4%; PN: 7% vs 0%; palmar-plantar erythrodysesthesia syndrome: 13.7% vs 0%	[61]
Aftimos 2016 (expanded access)	1.4 mg/m^2^; days 1, 8 q21d	154	Unknown	17%	Unknown	A, T, C; mean: 4; MCB regimen	All (24); ER+/HER2− (29); HER2+ (14); TNBC (21)	3.2 mo	11.3 mo	Neutropenia: 36.9%; FN: 9.2%; fatigue/asthenia: 9.2%; PN: 7.1%	[62]
Inoue 2016 (phase II)	1.4 mg/m^2^; days 1, 8 q21d	51	55 (33.9–74.4)	37.3%	T 100%	A (56.9%), T (98.1%), C (72.5%); mean: 2 (0–7) (after recurrence)	25.5	3.6 mo	11.7 mo	Leukopenia: 23.5%; neutropenia: 35.3%; anemia: 5.9%; FN: 7.8%	[63]
Maeda 2017 (phase II)	1.4 mg/m^2^; days 1, 8 q21d	24	Unknown	Unknown	NR	A, T ≥ 2 prior MBC regimen (21.3%)	8.3	3.5 mo	11.8 mo	Neutropenia: 45.8%; leukopenia: 29.1% FN 12.5%	[64]
Lorusso 2017 (sub analysis)	1.4 mg/m^2^; days 1, 8 q21d	91	62 (33–85)	9.9%	T 97.8%	≥2 prior chemotherapy	CR 2.2PR 17.6	3.1 mo	11.6 mo	Neutropenia: 12.1%; PN: 2.2%; asthenia: 4.4%	[65]
Ohtani 2018 (phase II)	1.4 mg/m^2^; days 1, 8 q21d vs days 1, 15 q28d	40 vs 42	61.0 (37–80) vs 60.0 (40–77)	42.5% vs 35.7%	Unknown	A, T; median: 2 prior regimens	20.0 vs 21.4	TTF: 75 vs 81.5 days	412 vs 523 days	Neutropenia: 22.5% vs 92.9%; leukopenia: 22.5% vs 95.2%; FN 2.5% vs 11.9; PN: 45% vs 52.4%	[66]

Ixabepilone											
Perez 2007 (phase II)	40 mg/m^2^ q21d	126	51 (30–78)	33%	Unknown	A, T, C ≥ 2 prior regimens (88%)	11	3.1 mo	8.6 mo	Neutropenia: 54%; PN: 14%; FN: <1%	[67]
Thomas 2007 (phase II)	50/40 mg/m^2^ q21d	49	54 (30–81)	37%	Unknown	T ≥ 2 prior regimens (31%)	12	2.2 mo	7.9 mo	Neutropenia: 53%; FN: 6%; PN: 12%	[68]
Aogi 2013 (phase II)	40 mg/m^2^ q21d	52	54.5 (30–76)	30.8%	T	A ≥ 2 prior regimens (91%)	11.5	2.8 mo	12.4 mo	Neutropenia: 83% FN: 6% PN: 19%	[57]
Smith 2013 (phase II)	40 mg/m^2^ q21d vs 16 mg/m^2^ days 1, 8, 15 q28d	91 vs 85	60.5 (37.4–79.8) vs 57.8 (39.1–81.2)	21.2% vs 24.2%	A T	mean 2 (range 0–7)	13.5 vs 7.6	5.3 vs 2.9 mo	16.1 vs 13.9 mo	Neutropenia: 38% vs 6%; FN: 2% vs 0%; PN: 16% vs 9%	[69]

FN, febrile neutropenia; HFS, hand-foot syndrome; MBC, metastatic breast cancer; NR, not reported; ORR, objective response rate; OS, overall survival; PFS, progression-free survival; PN, peripheral neuropathy; q14d, every 2 weeks; q21d, every 3 weeks; q28d, every 4 weeks; TPC, therapy of physicians' choice. TTF: time to treatment failure; PLD: pegylated liposomal doxorubicin.

**Table 3:** Newest agents.

Author (study phase)	Treatment	Patients (*n*)	Patients age (years); mean (range)		Triple-negative status	Drug resistance	Prior treatments	Efficacy			Safety grade (3/4AE)	References
								ORR (%)	PFS (months)	OS (months)		
Vinflunine												
Campone 2006 (phase II)	320 mg/m^2^ q3w	60	55.2 (33–75.8)	Unknown		Unknown	A, T	30	3.7	14.3	Neutropenia 65%, fatigue 16.7%, constipation 11.7%	[103]
Fumoleau 2009 (phase II)	320 mg/m^2^ q3w	56	54.3 (35.7–73.1)	Unknown		Unknown	A, T	12.5	2.6	11.4	Leukopenia 49.1%, fatigue 14.3%,constipation 7.1%, FN 5.4%	[104]
Blasinska-Morawiec 2012 (phase II)	320 mg/m^2^ q3w	38	56 (40–73)	Unknown		Unknown	Vinorelbine	8.3	4.0	13.6	Neutropenia 50%, leukopenia 34.2%, fatigue 15.8%	[105]
Cortes 2018 (phase III)	280 mg/m^2^ q3w vs alkylating agent	298 vs 296	58 (32–76) vs 57 (28–79)	19% vs 18%		A (58% vs 62%); T (84% vs 81%); antimetabolite (95% vs 92%); vinka alkaloids (100% vs 100%)	Mean regimen MBC 4 (2–12) vs 4 (0–13)	6 vs 13	2.5 vs 1.9 (*p*=0.49)	9.1 vs 9.3 (*p*=0.67)	Neutropenia 19% vs 11%, anemia5% vs 4%, asthenia 10% vs 4%	[106]
Irinotecan												
Hayashi 2013 (phase II)	150 mg/m^2^ d1, 15 q4w	18	59 (40–75)	38.9%		Unknown	A 83.3%; T 94.4%; A + T 77.8%	5.6	3.2	9.6	Neutropenia 22.2%, anorexia 11.2%, diarrhea 11.2%, fatigue 5.6%	[107]
Etirinotecan												
Awada 2013 (phase II)	145 mg/m^2^ q2w vs q3w	34 vs 35	53·0 (33–83) vs 56·0 (37–77)	31% vs 29%		Unknown	A 100%; T 100%; C 26% vs 26%; ≥2 prior MBC regimen: 49% vs 71%	29 vs 29	3.3 vs 5.6	8.8 vs 13.1	Neutropenia 11%; FN: <1%; diarrhea 21%	[108]
Perez 2015 (phase III)	Etirinotecan 145 mg/m^2^ q3w vs TPC	429 vs 423	55 (28–84) vs 55 (32–80)	28% vs 28%		A (14% vs 13%); T (42% vs 37%); C (71% vs 74%)	A 96%; T 100%; C 100%; mean previous regimen 3 (1–6)	16 vs 17	2.4 vs 2.8	12.4 vs 10.3	Neutropenia 10% vs 31%; diarrhea: 10% vs 1%; anemia 5% vs 5%; fatigue 4% vs 4%	[109]

## Data Availability

The data supporting this manuscript are extracted from the previously reported studies and datasets, which have all been cited.
